# Variation and risk factors of drug resistant tuberculosis in sub-Saharan Africa: a systematic review and meta-analysis

**DOI:** 10.1186/s12889-015-1614-8

**Published:** 2015-03-25

**Authors:** Deus Lukoye, Willy Ssengooba, Kenneth Musisi, George W Kasule, Frank G J Cobelens, Moses Joloba, Gabriela B Gomez

**Affiliations:** National Tuberculosis and Leprosy Program, Kampala, Uganda; Management Sciences for Health (MSH), Kampala, Uganda; Department of Medical Microbiology, Makerere University College of Health Sciences Kampala, Kampala, Uganda; Academic Medical Center, Department of Global Health and Amsterdam Institute for Global Heath and Development, Pietersbergweg 17, 1105 BM Amsterdam, The Netherlands; KNCV Tuberculosis Foundation, The Hague, Hague, The Netherlands; National TB Reference Laboratory, Kampala, Uganda; London School of Hygiene and Tropical Medicine, Department of Global Health and Development, London, UK

**Keywords:** Sub-Saharan Africa, Drug resistant tuberculosis, Risk factors, HIV, Survey

## Abstract

**Background:**

Prevalence of multidrug resistant tuberculosis (MDR-TB), defined as in vitro resistance to both rifampicin and isoniazid with or without resistance to other TB drugs, in sub-Saharan Africa (SSA) is reportedly low compared to other regions. These estimates are based on data reported to the World Health Organization (WHO) on drug resistance surveys, which may suffer from a reporting bias. We set out to evaluate the variation in prevalence of drug resistant tuberculosis (DR-TB) and its determinants across SSA countries among new and previously treated TB patients.

**Methods:**

The aim was to perform a systematic review and meta-analysis of DR-TB prevalence and associated risk factors in SSA. PubMed, EMBASE, Cochrane and bibliographies of DR-TB studies were searched. Surveys at national or sub-national level, with reported DR-TB prevalence (or sufficient data to calculate a prevalence) to isoniazid (INH), rifampicin (RMP), ethambutol (EMB), and streptomycin (SM) conducted in SSA excluding the Republic of South Africa, published between 2003 and 2013 with no language restriction were considered. Two authors searched and reviewed the studies for eligibility and extracted the data in pre-defined forms. Forest plots of all prevalence estimates by resistance outcome were performed. Summary estimates were calculated using random effects models, when appropriate. Associations between any DR-TB and MDR-TB with potential risk factors were examined through subgroup analyses stratified by new and previously treated patients.

**Results:**

A total of 726 studies were identified, of which 27 articles fulfilled the inclusion criteria. Studies reported drug susceptibility testing (DST) results for a total of 13,465 new and 1,776 previously treated TB patients. Pooled estimate of any DR-TB prevalence among the new cases was 12.6% (95% CI 10.6-15.0) while for MDR-TB this was 1.5% (95% CI 1.0-2.3). Among previously treated patients, these were 27.2% (95% CI 21.4-33.8) and 10.3% (95% CI 5.8-17.4%), respectively. DR-TB (any and MDR-TB) did not vary significantly with respect to study characteristics.

**Conclusions:**

The reported prevalence of DR-TB in SSA is low compared to WHO estimates. MDR-TB in this region does not seem to be driven by the high HIV prevalence rates.

## Background

Globally, the World Health Organization (WHO) reports an estimated prevalence of 3.6% and 20.2% among notified TB cases for primary and acquired multidrug resistant tuberculosis (MDR-TB), respectively, with significant country and regional variations [1]. Despite the high burden of TB in sub-Saharan Africa (SSA) fuelled by HIV [1], drug resistance surveillance has not been widely done, with only 22 of the 46 countries reporting drug resistance data by 2005. These studies have been designed to establish a nationwide MDR-TB prevalence only, and most of them had small sample sizes to assess variations between subpopulations or identify potential risk factors of the prevalence of drug resistance [2]. Yet, the use of inferior TB drug regimens, high HIV infection rates, and a wide roll-out of ART may predispose countries in this region to high levels of drug resistant tuberculosis (DR-TB) [3]. In particular, previous exposure to anti-TB treatment is a well-established risk factor for DR-TB [4]. By 2010, a number of TB programs in SSA were still using the eight-months regimen of two months of ethambutol (EMB), isoniazid (INH), rifampicin (RMP), and pyrazinamide (PZA), followed by six months of EMB and INH. This regimen has been associated with lower cure rates and higher rates of relapse than the currently recommended six-months regimen in which rifampicin is given throughout treatment (two months of EMB,INH,RMP,PZA followed by four months of RMP and INH) [4]. Conversely, duration of RMP treatment beyond four months has been associated with increased risk of acquiring drug resistance in initially drug sensitive strains [5]. Additionally, there have been concerns that, in SSA, six months of directly observed therapy are often unfeasible, and RMP throughout would increase the incidence of MDR-TB, in particular in the context of high HIV prevalence and pre-existing INH resistance [6]. While some drug resistance studies have shown an association between HIV and DR-TB/MDR, data showing HIV as an independent risk factor for MDR-TB in individuals have been limited to particular settings [7]. Nevertheless, high mortality among HIV patients suffering from MDR or extensively drug-resistant tuberculosis (XDR: defined as resistance to any of the fluoroquinolones (such as ofloxacin or moxifloxacin) and to any of the three injectable second-line anti-TB drugs (amikacin, capreomycin, or kanamycin) in addition to MDR) [8] are major concerns to TB control programs in SSA. Finally, the association between RMP mono-resistance and HIV infection has also been documented [9]. Therefore, understanding the role of potential ‘drivers’ of DR-TB in SSA is important to guide intervention policies and future drug resistance monitoring in the region. We did a systematic review and meta-analysis of published and unpublished studies to establish the variation of DR-TB across SSA countries and its determinants.

## Methods

### Data sources

We searched PubMed, EMBASE, and Cochrane for original publications from 2003 to 2013 without language limitations. Search terms used included *anti-TB drug resistance, drug resistant tuberculosis, M/DR/XDR-TB, and (isoniazid or rifampicin or ethambutol or streptomycin or ofloxacin or fluoroquinolone or kanamycin or amikacin) resistance* for each country in SAA, excluding the Republic of South Africa (RSA). Each term was searched separately with a text string ending with the specific name of the country. We excluded RSA because drivers of DR-TB in this country are likely to be different and prevalence has been reported to be substantially higher than the rest of SSA countries [10]. We also searched bibliographies of other reviews and citations of the original articles identified*.* Reviewers obtained unpublished DR-TB studies through personal communication with experts and authors of papers identified.

### Study selection

We included surveys carried out both at national or sub-national level reporting M/DR-TB prevalence or sufficient data to calculate a prevalence of resistance to isoniazid (INH), rifampicin (RMP), ethambutol (EMB), streptomycin (SM), and/or MDR (INH and RMP). Conference proceedings, chapters of books, and correspondences were excluded. Studies were considered of sufficient quality for inclusion if participants were classified as new or previously treated based on the WHO definition [11], the study covered a large geographical area (district, region, or entire country), and recommended laboratory procedures for culture and drug susceptibility testing (DST) were followed [12]. Studies conducted in a single health unit e.g. a referral hospital or a TB center, or those where fewer than 50 participants had DST were excluded to minimize bias of including non-representative samples of the population. Where cluster sampling was used, adjustment for the cluster design was a requirement for inclusion in this review.

Two authors conducted the electronic searches independently; the last search was conducted in June 2014. Selection of articles was done by both reviewers independently. Disagreements on articles to be included were resolved by consensus among the two authors.

### Data extraction

We extracted data using pre-defined forms on: country of the study; sampling method; description of the facilities where the study was done; total number of patients enrolled in the study as per treatment category; number of patients with DST results; number of patients with a positive result for resistance to INH, RMP, EMB, SM, or MDR-TB; and HIV prevalence among the participants (if available). HIV prevalence at national level for each country of interest was collected from the UNAIDS report 2013 [13]. Two authors extracted data independently and any discrepancies in the data extracted were resolved through discussions.

### Data synthesis and analysis

According to WHO, resistance among new cases is defined as resistance to one or more anti-tuberculosis drugs in patients that have never been treated for TB. Resistance among previously treated TB patients, on the other hand, is defined as resistance to one or more anti-tuberculosis drugs in patients that have been treated for TB. It can be transmitted from another patient with DR-TB or acquired in patients diagnosed with pan-sensitive TB who have started TB treatment and subsequently develop resistance to one or more of the drugs used during the treatment. To generate data stratified for the resistance among the new and previously treated TB patients, we calculated pooled resistance prevalence along with the 95% confidence interval through meta-analysis using random effects models for MDR-TB and any DR-TB to the first line drugs (INH, RMP, EMB, and SM). We assessed the heterogeneity among reported prevalence using the *I*^*2*^ statistic.

To explore the variation observed in the prevalence estimates, we did a subgroup analysis by stratifying studies by predefined variables. In particular, we categorized variables as follows: 1) by sub-region (Eastern sub-region included Burundi, Ethiopia, Kenya, Rwanda, Somalia, Uganda, and Tanzania; West Africa sub-region included Benin, Burkina Faso, Cameroon, Equatorial Guinea, Gambia, Ghana, Ivory Coast, and Nigeria; Southern sub-region: Botswana, Zambia, Mozambique, Madagascar, Swaziland, and Zambia; and Central Africa sub-region: Central African Republic and Chad); 2) HIV prevalence at a national level (countries with a prevalence of less than 5% compared to those with a prevalence of more than 5% in the general population); 3) type of survey (national or sub-national); 4) sampling method (random sampling or cluster sampling); 5) sample size (studies of less than 100 patients or more than 100 patients); and HIV prevalence among study participants (less than 40% compared to, equal to or more than 40%).

We avoided use of acquired resistance for these categories of patients due to limitations of this definition for acquired resistance as it does not put into consideration possibilities of re-infection with resistant forms and initial infection with resistant strains contributing to treatment failure, since capacity to ascertain resistance patterns prior to treatment initiation is rarely available under routine settings.

## Results

**Figure 1:**
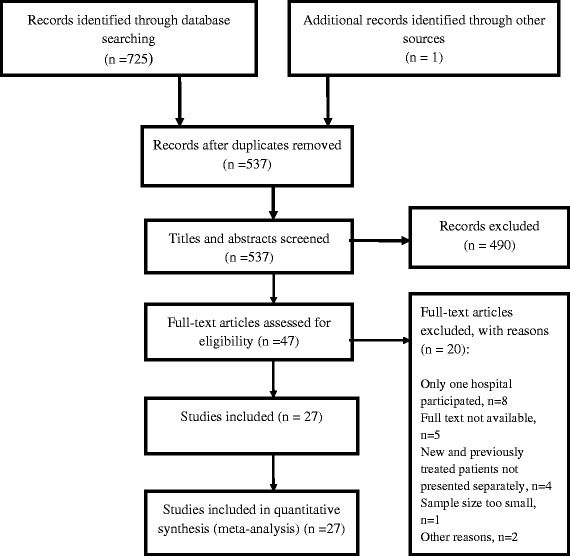
**PRISMA flow chart.**

We identified 725 citations through electronic data searches and one completed study with unpublished data. Out of these, 47 articles were selected for full text review, of which 20 articles were excluded for various reasons (Figure 1). Characteristics of the 27 articles included are summarized in Table 1. Of these 27 studies, 19 (70%) reported DR-TB data on both new and previously treated patients. Seven studies reported resistance among new cases only, while one study assessed DR-TB among the previously treated. Sixteen (59%) studies reported HIV testing, and HIV prevalence estimates at country level were available for more than 90% of the studies. Thirteen (48.1%) studies in total reported data at national level. Compared to other regions, the eastern region contributed the highest number of articles, five of which were from national surveys.

**Table 1:** **Characteristics of studies included in the review of variation of M/DR-TB in SSA; 2003–2013**

**Author**	**Study year**	**Country**	**Study description**	**Patient category**	**Sample size (included in DST)**	**HIV prevalence in the study (%)**	**Country HIV prevalence (%)**	**DST method**	**Type of resistance tested**
Minime-Lingoupou F *et al.* [21]	2009	Central African Republic	Sub-national survey. TB health facilities in Bangui and Bimbo.	New patients	233	26	N/A	LJ	INH, RMP, SM, EMB
Asmamaw D. *et al.* [22]	2004	Ethiopia	Sub-national survey. Twenty-four TB health facilities in Addis Ababa.	New patients	231	29.6	2.9	LJ	INH, RMP, SM, EMB
Abdelhadi O. *et al.* [23]	2009-2010	Chad	Sub-national survey. Number of TB facilities not provided.	New patients	135	25	3	LJ	INH, RMP, SM, EMB
Yimer S.A. *et al.* [24]	2008	Ethiopia	Sub-national survey. Number of TB facilities in Amhara not provided.	New patients	112	26.9	1.9	MGIT	INH, RMP, SM, EMB
Urassa W. *et al.* [25]	2001-2004	Tanzania	Sub-national survey. Five TB health facilities in Dar es Salaam.	New patients	887	53	5.7	LJ	INH, RMP, SM, EMB
Ndungu PW. *et al.* [26]	2010	Kenya	Sub-national survey. Five TB health facilities in and around Nairobi.	New patients	356	26.3	6.6	MGIT/LJ	INH, RMP, SM, EMB
Matee M. *et al.* [27]	2005-2006	Tanzania	Sub-national survey: Thirty-seven TB facilities of Temeke district.	New patients	226	N/A	5.8	LJ	INH, RMP, SM, EMB
Lukoye D. *et al.* (a) [28]	2008	Uganda	Sub-national survey. Twenty-two TB health facilities in Kampala.	New and PT patients	557	30.9	6.7	LJ	INH, RMP, SM, EMB. Km and O
Sanders M. *et al.* [29]	2008	Burundi	Sub-national survey. Seven TB health facilities in Bujumbura.	New and PT patients	859	N/A	2.2	LJ	INH, RMP, SM, EMB, PABA
Lukoye D. *et al.* (b) [30]	2009-2011	Uganda	National survey.	New and PT patients	1537	30.7	7.3	LJ	INH, RMP, SM, EMB, KM and OFX
Umubyeyi A. N. *et al.* [31]	2004-2005	Rwanda	National survey.	New and PT patients	701	N/A	3.3	LJ	INH, RMP, SM, EMB
Irenious S. *et al. *[15]	2011	Somalia	National survey.	New and PT patients	946	N/A	N/A	Hain	INH, RMP only
Chonde TM *et al. *[32]	2006-2007	Tanzania	National survey.	New and PT patients	1,167	N/A	5.8	LJ	INH, RMP, SM, EMB
Tessema B. *et al. *[33]	2009	Ethiopia	Sub-national survey. Five TB health facilities in north west Ethiopia.	New and PT patients	260	25.4	1.7	LJ	INH, RMP, SM, EMB, CPM, OFX, AM, MFX, Amino Salicylic Acid
Chanda M. *et al.* [34]	2006	Zambia	Sub-national survey. Six TB health facilities in Ndola district.	New and PT patients	361	N/A	13.2	LJ	INH, RMP, SM, EMB
Nunes E.A. *et al.* [35]	2002-2003	Mozambique	Sub-national survey. Number of TB health facilities not provided.	New and PT patients	111	N/A	9.8	LJ	INH, RMP, SM, EMB
Nelson L.J. *et al.* [36]	2002	Botswana	National survey.	New and PT patients	2,425	60	25.7	LJ	INH, RMP, SM, EMB
Ramarokoto H. *et al. *[37]	2005-2007	Madagascar	National survey.	New and PT patients	1,275	N/A	0.6	LJ	INH, RMP, SM, EMB
Samo Gudo P. *et al.* [38]	2007-2008	Mozambique	National survey.	New and PT patients	1,200	N/A	11.5	LJ	INH, RMP, SM, EMB
Sanchez-Padilla E. *et al.*	2009	Swaziland	National survey	New and PT patients	633	79.9	25.8	MGIT or LJ	INH, RMP, SM, EMB
Edgbola R.A. *et al.* [39]	1999	Gambia	National survey.	New and PT patients	225	N/A	2.1	LJ	INH, RMP, SM, EMB
Affolabi D. *et al.* [40]	2002-2004	Benin	Sub-national survey. National Pneumo-Phthisiology hospita receiving patients from Benin and surrounding countries	New and PT patients	470	10.2	2.3	LJ	INH, RMP, SM, EMB
Tudo G. *et al.* [41]	2004	Equatorial Guinea	Sub-national survey. Number of TB health facilities not provided.	New and PT patients	236	13.5	3.6	LJ	INH, RMP, SM, EMB
N'guesesan K. *et al. *[42]	2005	Ivory Coast	National survey.	New patients	320	N/A	4.9	LJ	INH, RMP, SM, EMB
Sangare L. *et al.* [43]	2010	Bukina Furso	National survey.	New and PT patients	416	28.7	1.3	LJ	INH, RMP, SM, EMB
Ellis Awusu-Dabo *et al.* [44]	2001-2004	Ghana	National survey.	New and PT patients	216	25.9	4.7		INH, RMP, SM, EMB, Thiacetazone
Jurgen Noesk *et al.* [45]	2012	Cameroon	Sub-national. Twenty-nine TB health facilities in Litoral region.	PT patients	233	26	N/A	LJ	INH, RMP, SM, EMB, Km and GFX
Mbulo G.M.K. *et al.* Results of the national drug resistance survey in Zambia. (in preparation)	2008	Zambia	National survey.	New and PT patients	883	47.6	13.3	LJ	INH, RMP, SM, EMB

*Abbreviations*: *DST* drug susceptibility testing, *INH* isoniazid, *RMP* rifampicin, *EMB* ethambutol, *SM* streptomycin, *Km* kanamycin, *GFX* gatifloxacin, *CPM* capreomycin, *OFX* ofloxacin, *AM* amikacin, N/A, not available, *LJ* Löwenstein Jensen, *MGIT* mycobacteria growth indicator tube, *PT* previously treated patient.

**Figure 2:**
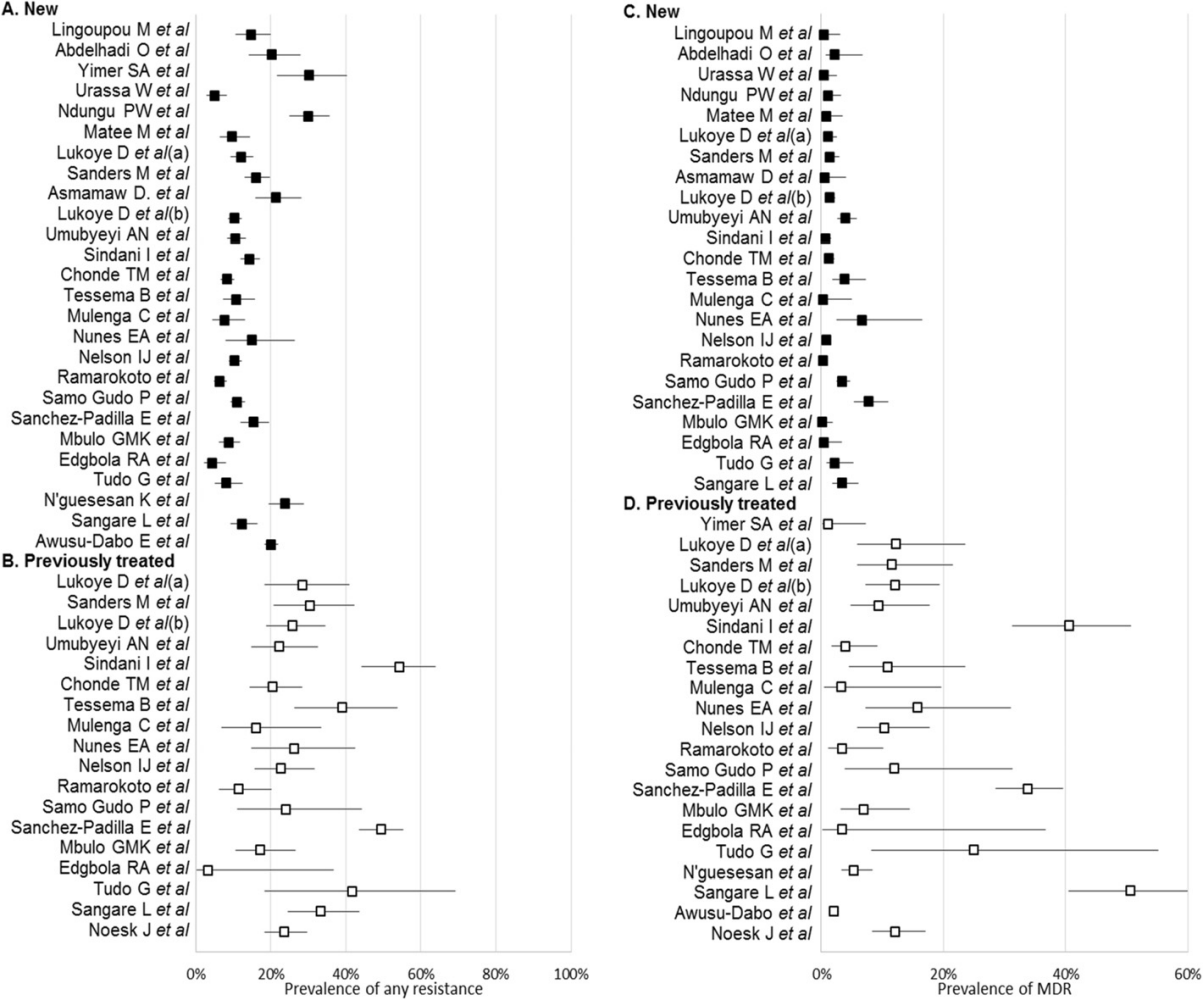
**Forest plot of prevalence of any resistance and MDR among new and previously treated patients.**

**Figure 3:**
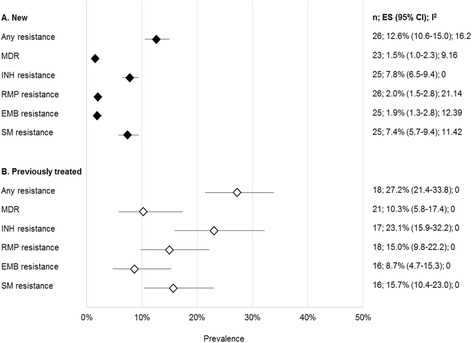
**Pooled estimates for all resistance patterns among new and previously treated patients.** Abbreviations: n, number of studies; ES, estimate; INH, isoniazid; RMP, rifampicin; EMB, ethambutol; SM, streptomycin.

DR-TB data was reported for a total of 15,462 sputum smear-positive TB patients in the 27 articles included from 2003 to 2013. Of these, 13,645 (88.4%) and 1,776 (11.6%) were new and previously treated patients, respectively. All reported estimates for any resistance and MDR-TB among new and previously treated patients are presented separately by study in Figure 2. In Figure 3, we then present pooled estimates for all resistance patterns, including MDR-TB among new and previously treated patients. Prevalence of any DR-TB and of MDR-TB were higher among patients who had been previously treated for TB (Figures 2 and 3). Overall, the pooled prevalence of any DR-TB among new and previously treated patients was 12.6% (95% CI 10.6-15.0%) and 27.2% (95% CI 21.4-33.8), respectively; while MDR-TB among the new and previously treated patients was 1.5% (95% CI 1.0-2.3) and 10.3% (95% CI 5.8-17.4), respectively. Summary estimates for any DR-TB among new and previously treated TB cases were highest for INH [7.8% (95% CI 6.5-9.4) and 23.1% (95% CI 15.9-32.2)] and lowest for EMB [1.9% (95% CI 1.3-2.8) and 8.7% (95% CI 4.7-15.3)] (Figure 3). Resistance to RMP in new cases, 2.0% (1.5-2.8) was also very low (Figure 3).

### Variation of DR-TB with key study characteristics

**Figure 4:**
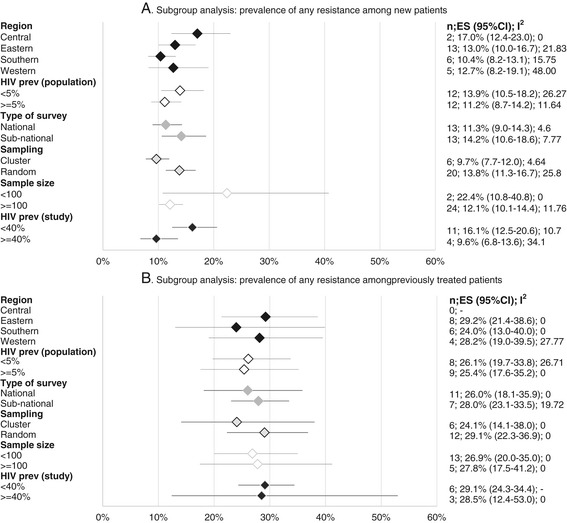
**Subgroup analysis: prevalence of any drug resistance. Abbreviations: n, number of studies; ES, estimate.** Where data is missing, it means that such a region did not have any study fitting that classification for inclusion in the analysis.

**Figure 5:**
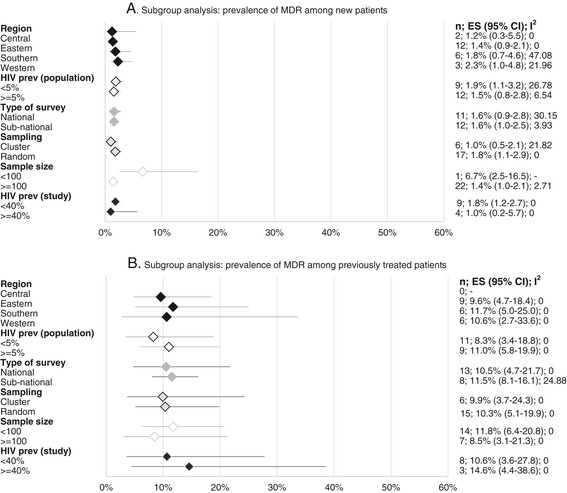
**Subgroup analysis: prevalence of MDR-TB.** Abbreviations: n, number of studies; ES, estimate, MDR- TB, Multi-drug resistant tuberculosis. Where data is missing, it means that such a region did not have any study fitting that classification for inclusion in the analysis.

In Figures 4 and 5, we present the subgroup analyses for the prevalence of any DR-TB and MDR-TB by study characteristics. Overall, we observed larger variations in the pooled estimates by subgroup with respect to any DR-TB, compared to MDR estimates.

#### Regional variations

Prevalence of any DR-TB among new cases varied from 10.4% (95% CI 8.2- 13.1, n = 6) in the Southern region to 17.0% (12.4-23.0, n = 2) in the Central region. Any DR-TB among previously treated TB patients was highest in East Africa with levels of 29.2% (95% CI 21.4-38.6, n = 8) and lowest in the Southern African countries, 24.0% (95% CI 13.0-40.0, n = 6). MDR TB among new patients was lowest in Central Africa at 1.2% (95% CI 0.3-5.5, n = 2) and highest in Western Africa, 2.3% (95% CI 1.0-4.8, n = 3), while MDR-TB among previously treated was highest in Southern region, 11.7% (95% CI 5.0-25.0, n = 6) and lowest in Eastern region, 9.6% (95% CI 4.7-18.4, n = 9). We did not observe significant variations in pooled estimates of any DR-TB or MDR-TB in the sub-regions as shown by the overlap in the 95% CIs of our estimates (Figures 4 and 5).

#### Country-level HIV prevalence

Analysis of any DR-TB among new cases in relation to HIV infection rates (Figure 4) showed somewhat higher resistance rates of 13.9% (95% CI 10.5-18.2, n = 12) in countries where HIV prevalence was lower than 5%, compared to countries where the prevalence was equal to or higher than 5%, [11.2% (95% CI 8.7-14.2, n = 12)], while DR-TB among the previously treated was almost the same among settings with these different HIV prevalence rates (26.1%, n = 8 vs 25.4%, n = 9). Primary MDR-TB in settings with less than 5% HIV prevalence was 1.9% (95% CI 1.1-3.2 n = 9) as compared to 1.5% (95% CI 0.8-2.8 n = 12) in settings where the HIV prevalence equal to or higher than 5%. MDR-TB among previously treated patients in countries with lower than 5% HIV prevalence was 8.3% (95% CI 3.4-18.8, n = 11) compared to 11.0 (95% CI 5.8-19.9, n = 9) in countries with HIV prevalence of equal to or higher than 5%. However, differences were small with largely overlapping 95% confidence intervals.

#### TB/HIV co-infection

Where HIV testing was done as part of the survey (Figure 4), we observe a higher prevalence of DR-TB among new cases in studies where HIV was lower than 40% among the study participants [16.1% (95% CI 12.5-20.6, n = 11)] as compared to 9.6% (95% CI 6.8-13.6, n = 4) in studies where HIV prevalence among participants was equal to or higher than 40%. Analysis of DR-TB among previously treated cases in relation to these HIV co-infection rates shows the same rates in these two settings, those studies with lower than 40% HIV co-infection and equal to or more than 40% of HIV co-infection, [29.1%, 95% CI = 24.3-34.4 n = 6 and 28.5% 95% CI 12.4-53.0 n = 3]. MDR among new cases in studies where TB/HIV co-infection rates were lower than <40% was 1.8% (1.2-2.7, n = 9); and 1.0% (0.2-5.7; n = 4); in studies with equal to or higher than 40% TB/HIV co-infection. MDR-TB among previously treated patients where TB/HIV co-infection was lower than 40% among the participants was 10.6% (95% CI = 3.6-27.8, n = 8) and 14.6% (95% CI 4.4-38.6, n = 3) where equal to or higher than 40% of the participants were HIV co-infected, although this difference was also not significant (Figure 5).

#### Study geographical coverage

Generally, articles reporting national surveys estimated lower rates of any DR-TB among new cases 11.3% (95% CI = 9.0-14.3, n = 13) as compared to sub-national reports 14.2% (95% CI 10.6-18.6, n = 13). Any acquired DR-TB was similar in the national (26.0%; 95% CI 18.1-35.9, n = 11) and sub-national surveys (28%; 95% CI- 23.1-33.5, n = 7) (Figure 4). MDR estimates among new cases were the same in both national and sub-national studies at 1.6% (95% CI 0.9- 2.8, n = 11) and 1.6% (95% CI 1.0-2.5, n = 12) respectively, as were MDR rates among the previously treated: 10.5% (95% CI 4.7-21.7, n = 13) versus 11.0% (95% CI 5.8-19.9, n = 8), respectively (Figure 5).

#### Sampling design

Studies that applied a cluster sampling design reported lower rates of any DR-TB 9.7% (7.7-12.0; n = 6) in new cases than studies where random sampling was used 13.8% (11.3-16.7; n = 20); DR-TB rates among previously treated patients in these two study designs were 24.1% (14.1-38.0; n = 6) and 29.1% (22.3-36.9 n = 12) respectively. Rates of MDR-TB followed a similar trend with MDR-TB among the new patients in studies that used cluster and random sampling designs reporting MDR-TB rates of 1.0% (0.5-2.1; n = 6) and 1.8% (1.1-2.9; n = 17), respectively. MDR-TB among the previously treated category in studies that used cluster design was 9.9% (3.7-24.3; n = 6), similar to that in studies where random sampling was used [10.3% (5.1-19.9; n = 15)]. All the differences in these measurements did not show statistical significance (Figures 4 and 5).

#### Sample size

Studies with sample sizes of less than 100 participants reported significantly higher rates of any DR-TB among new cases, 22.4% (95% CI 10.8-40.0, n = 2) compared to studies where 100 or more participants were recruited, 12.1% (95% CI 10.1-14.4, n = 24). Levels of DR-TB among the previously treated were almost the same in both categories of sample size, 26.9% (95% CI 20.0-35.0, n = 13) and 27.8% (95% CI 17.5-42.1, n = 5). For either category of study size, MDR levels amongst new cases followed similar trends, significantly higher 6.7% (95% CI 2.5-16, n = 1) in studies with less than 100 participants as compared to 1.4% (95% CI 1.0-2.1, n = 22) in studies with larger sample sizes. Although slightly higher, levels of MDR-TB among previously treated patients in studies with less than 100 participants, 11.8% (6.4%-20.8%, n = 14), this difference was not statistically significant as compared to studies with 100 participants or more, 8.5% (95% CI 3.1%-21.3%, n = 7).

### Publication bias

**Figure 6:**
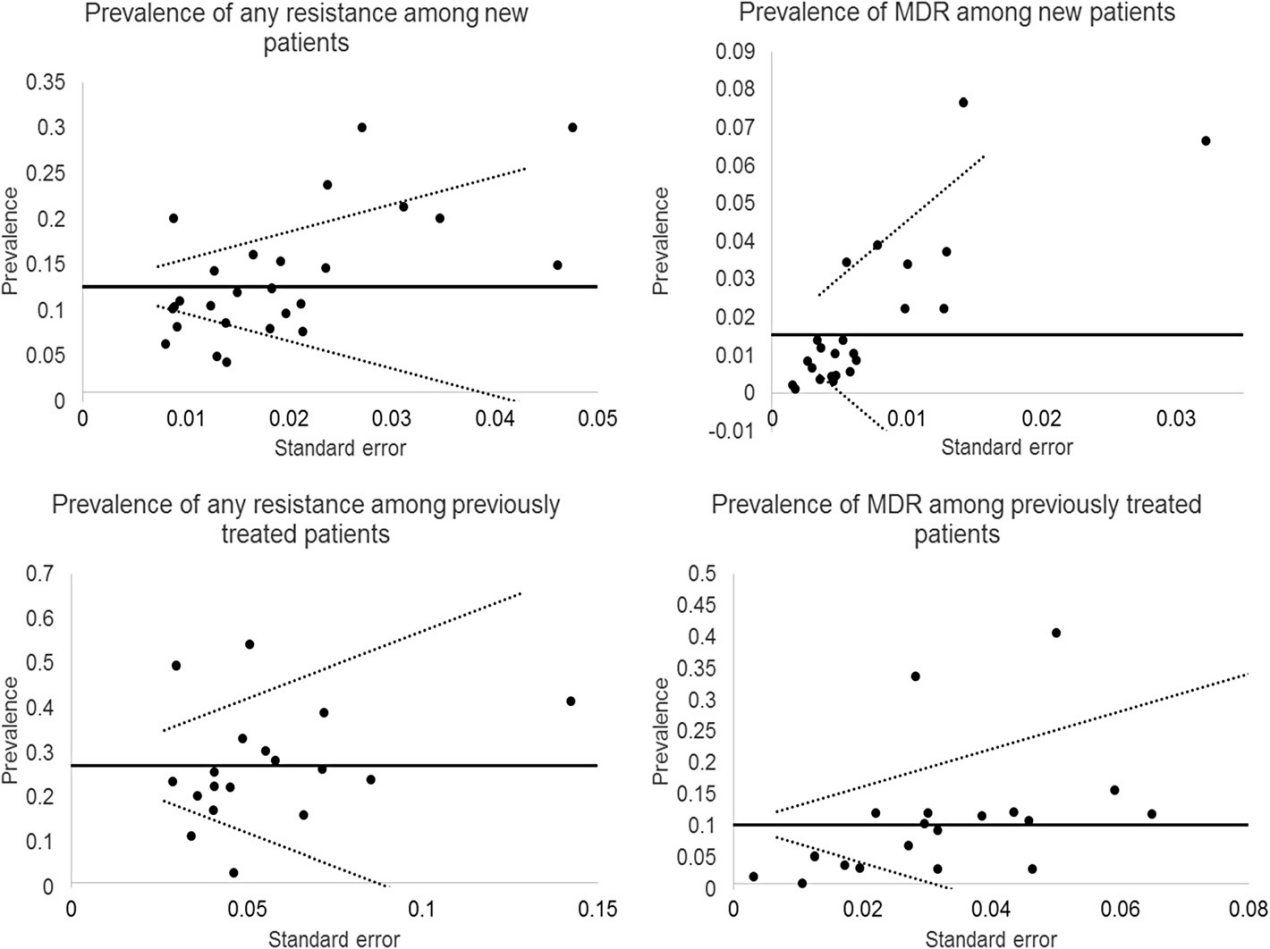
**Funnel plot exploring publication bias.** The horizontal line represents the summary prevalence and guidelines are given to indicate the 95% confidence interval for this estimate.

Finally, in Figure 6, we explored graphically the possibility of a publication bias. We did not observe an indication of such a bias in the studies included.

## Discussion

In our study, we reviewed variations and risk factors of DR-TB in SSA. We found that levels of any DR-TB and MDR-TB are lower in SSA than reported globally [1]. In particular, our results show MDR-TB prevalence estimates as almost half as compared to the global average reported by WHO for both new (1.5% vs 3.6%) and previously treated TB patients (10.3% vs 20.2%) [2]. These consistent low levels occur in settings with high rates of HIV, largely attributed, among other factors, to the late introduction of RMP and limited availability of TB drugs on the open market outside national TB programs [14] in this region. According to the subgroup analyses, rates of (M)DR-TB remain generally low regardless of the study geographical coverage, sample size, HIV co-infection rates, and sub-region where the study was conducted. This finding happens at a time when more information on rates and factors associated with of DR-TB in this region is emerging, as more countries conduct surveys at national and sub-national level [14], although data on DR-TB from SSA is still limited [10]. The observed low levels of (M)DR-TB may also reflect the functionality of TB control programs in this region. Previous studies have shown that countries where standardized regimens are available and properly implemented, where quality drugs are regularly supplied, and where systems are in place to ensure patients’ adherence are less likely to report high rates of (M)DR-TB. From our findings, such explanation can be supported by the high rates of MDR-TB from the Horn of Africa included in our review, which could have resulted from a break down in the public health system and therefore in the functionality of the TB program due to civil strife also observed elsewhere in the world [16,17]. Therefore, regional variations in MDR-TB rates might be considered a proxy measure for functionality of national TB programs which should alert national governments and donor communities for timely interventions. The role of *Mycobacterium tuberculosis* (MTB) strains in transmissibility and its potential to develop DR in this region should not be ignored. As observed in some settings, particular MTB strains predominant in specific localities have been associated with varying rates of MDR-TB [18]. Hence, more molecular studies are required to examine and explain possible associations of the predominant MTB strains with the observed prevalence of DR-TB in SSA. Our findings seem to imply that transmission-related factors such as late diagnosis, nosocomial spread, and delay in initiation of second-line treatment as observed in most settings of this region have not led to increase in (M)DR-TB above the minimum WHO estimates. However we observe higher rates of resistance to INH and SM than other drugs in our analysis, also documented earlier, attributed to the long history of INH and SM use in management of TB and to the stepwise acquisition of DR by MTB to these two drugs [19].

Lower levels of MDR-TB (1.5%) in settings with higher HIV prevalence at population level, also observed where HIV testing was included in the study design, could result from less participation rates of (M)DR-TB/HIV co-infected patients in surveys due to either severe illness or higher risk of death [8]. Where collection of individual HIV data was included in the study design, we found higher rates of MDR-TB (25%) among previously treated patients in studies where HIV prevalence was lower, possibly due to the same explanation and the possibility of suspected high MDR-TB rates in such populations.

We observed levels of any RMP resistance among new cases (1.5%) in the analysis close to the reported prevalence of MDR-TB (2.0%). This finding is of significant relevance in the current global and regional efforts to accurately and timely diagnose MDR-TB with the scale-up of molecular technology like GeneXpert MTB/RIF, providing quick results of RMP resistance as a proxy to MDR-TB. In fact, in many SSA countries, access to culture and DST facilities is limited and molecular technologies might ease access to MDR-TB diagnosis and reduce the time spent between diagnosis and initiation of the patient on treatment. High levels of INH and SM resistance found in our review, also documented elsewhere, need to be monitored closely in relation to the potential increase in treatment failure and relapse rates with the current first-line drugs [20]. In light of the recommended roll-out of the RMP-through regimen by WHO, especially in high HIV burden settings such as SSA, TB programs need to ensure correct use of RMP in drug -susceptible cases to avoid adding RMP resistance to the already high levels of INH resistance, likely to lead to high MDR-TB rates.

Finally, we observe higher rates of MDR-TB in smaller studies as compared to larger ones possibly arising from the difference in the core objectives of the studies. Studies with small sample sizes are usually done to explore possibilities of high MDR-TB rates in specific populations. Similarly, DR-TB rates in sub-national studies are higher than in the national surveys since, in most cases, sample sizes in such studies tend to be smaller, non-representative of the population, and sometimes do not apply standardized methodologies, although we aimed to exclude such studies from our analysis. The lower (M)DR levels observed in cluster surveys as compared to surveys where random sampling was applied may have a similar explanation. Cluster sampling designs are usually applied where the study population is large and covering a wider geographical area for optimal use of resources without compromising the quality of the data. Consequently, lower (M)DR rates in cluster surveys could have been a proxy to the large sample sizes involved.

As demonstrated by the publication bias sub-analysis, we observed no tendency from authors to publish papers showing more or less resistance more frequently that could distort our findings.

### Limitations

Our review had some limitations. Of 44 countries in SSA (excluding the Republic of South Africa), only 20 countries had done studies that fulfilled our inclusion criteria, of which studies from five countries were not on a national scale. Many of the DR-TB surveys identified during our searches were excluded because they took place at a single health facility or had not stratified patients according to their treatment history.

Although the association between HIV infection and DR-TB is still controversial and deserves further exploration, ten of the 27 studies analyzed did not include HIV testing. It was, therefore, difficult to draw meaningful conclusions. Similarly, we did not review data on national ART coverage due to challenges associated with accessing accurate data to examine a possible relationship between ART roll-out and levels of MDR-TB. Finally, results on second-line DST were not reported for the majority of studies. This could be a reflection that most countries in SSA had not initiated MDR-TB treatment at the time of the study and the possibilities of finding XDR-TB were limited, although this analysis would be important especially in settings where some fluoroquinolones (a cornerstone of second-line drug regimens) are widely used for treatment of other bacterial infections.

We excluded the republic of South Africa on the basis of high levels of MDR-TB and XDR-TB rates in comparison to other countries of SSA [1,8], possibly fuelled by high nosocomial transmission rates in the context of very high rates of TB/HIV co-infection reported in this country. We assumed that including such studies could potentially skew our results towards higher DR-TB or MDR-TB estimates.

## Conclusions

Our analysis showed low levels of MDR-TB in sub-Saharan Africa compared to WHO estimates, with higher resistance to INH and SM as reported elsewhere in the world. There are no major variations in MDR-TB burden by sub-region and evidence of association between MDR-TB and HIV infection rates did not show statistical significance. We attribute these low levels to the limited existence of anti-TB drugs outside the national programs, late introduction of RMP in SSA, and wide use of fixed drug combinations. Since these factors may apply to other settings where rates of MDR-TB are higher, more studies are required to explore other possible explanations for the low levels of MDR-TB in SSA, such as the role of predominant MTB strains in generation and transmission of DR-TB in this region

## Abbreviations

ART: Anti-retroviral therapy
DR-TB: Drug resistant tuberculosis
DST: Drug susceptibility testing
EMB: Ethambutol
INH: Isoniazid
MDR-TB: Multi Drug Resistant Tuberculosis
RMP: Rifampicin
RSA: Republic of South Africa
SM: Streptomycin
SSA: Sub-Saharan Africa
TB: Tuberculosis
WHO: World Health Organization
XDR- TB: Extensively drug resistant tuberculosis
PZA: Pyrazinamide
